# A low-density cellulose rich new natural fiber extracted from the bark of jack tree branches and its characterizations

**DOI:** 10.1016/j.heliyon.2022.e11667

**Published:** 2022-11-17

**Authors:** Shahin Hossain, M. Abdul Jalil, Tarikul Islam, Md Mostafizur Rahman

**Affiliations:** a Department of Environmental Science, BGMEA University of Fashion and Technology (BUFT), Dhaka 1230, Bangladesh; b Department of Natural Sciences, BGMEA University of Fashion and Technology (BUFT), Dhaka 1230, Bangladesh; c Department of Textile Engineering, Jashore University of Science and Technology, Jashore-7408, Bangladesh; d Pulp and Paper Research Division, Bangladesh Council of Scientific and Industrial Research (BCSIR), Dhaka 1205, Bangladesh

**Keywords:** Natural fiber, Cellulose, Jack tree, Crystallinity, Water retting

## Abstract

The demand of natural cellulosic fibers has been increasing day by day due to their versatile uses and eco-friendly nature. The reason behind this demand is due to some unique properties of natural fibers that are suitable for several fibrous applications such as in composite, textile, nano-materials, conductive carbon, biomaterials etc. In this study, a new natural cellulosic fiber is extracted from the bark of the jack tree branches by water retting process. The fiber is characterized by standard methods. The result of the chemical compositions of the fiber shows that it contains α-cellulose 79.32%, hemicellulose 8.01%, lignin 6.77%, ash 3.58% and extractives 2.32%. XRD analysis reveals its high level of crystallinity (86%) and the microfibrillar angle (MFA) calculated from the XRD data is found −29°. The FTIR analysis confirms the presence of expected functional groups. Thermogravimetric analysis (TGA) and the derivative thermogravimetric (DTG) reveal its good thermal stability and the maximum degradation occurred at 358 °C for the degradation of the α-cellulose. The density of the fiber is found 1.05 g/cc, which is lower compared to many other known natural fibers. All these properties of this new fiber are suitable for several sophisticated fibrous applications such as reinforcement in composite, textile, cellulose nano-materials, activated or conductive carbon, biomaterials etc.

## Highlights

• The bark of Jack tree branches is a potential new source of high-quality fiber.
• The α-cellulose content (79.32%) and the crystallinity level (86%) of the fiber are comparatively high.
• The density of the fiber (1.05 g/cc) is comparatively low however, its thermal stability (358 °C) is high.
• The source of the fiber is reproducible and can be collected periodically for many years.

## Graphical abstract

**Figure:**
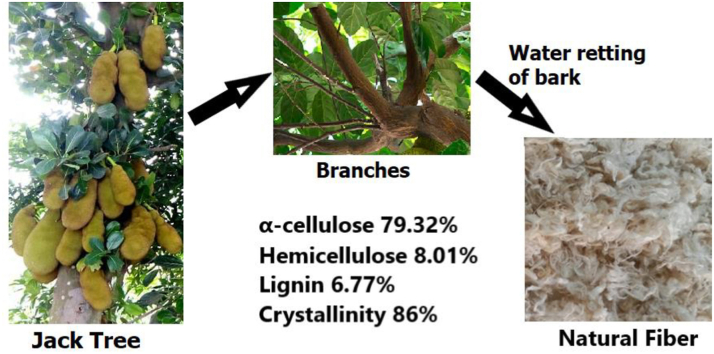

## 1 Introduction

In recent years, interest in natural cellulosic fiber has been growing continuously and the researchers from both academia and industries are now actively engaged in exploring new natural fibers and their new applications. The main reason of this interest is linked to their specific properties that are suitable for several sophisticated fibrous applications such as reinforcement in composite, textile, cellulose nano-materials, activated or conductive carbon, biomaterials etc. The unique properties which exist in natural fibers are their low density, low cost, availability, recyclability, non-toxicity, considerable strength, good thermal stability, bio-degradability etc. compared to their counterpart synthetic fibers [1, 2, 3, 4]. Hence, it is very important to explore new natural sources of cellulosic fiber because of their huge demand in fibrous applications.

Recently, research on natural cellulose-based materials has greatly increased in order to explore their new applications [5, 6]. Natural fiber reinforcement in composites is better than the synthetic fiber because of some special properties such as light weight, ecofriendly, easy fabrication etc. These reinforced composites have already been used successfully in various sectors such as household appliances, automobile, aerospace, marine, construction industries etc. Similarly, there is also a huge demand of innovative bio-based materials. Plants provide an excellent, cheap and abundant source of cellulosic biopolymer which are being used for the production of ecofriendly, green, profitable and sustainable functional materials.

The natural cellulosic fibers are separated from various parts of the plants such as leaf, root, bark, fruit, stem, stalk etc. Extracting fiber from plants is cost effective, harmless as well as the used plants/parts are reproducible. Some common and widely used natural fiber are flax, hemp, jute, kenaf, sisal, ramie, coir, pineapple leaf etc. However, the present production scale of cellulosic fibers cannot fulfill the growing demand from industries and therefore, it is very important to explore more new sources for natural cellulosic fibers. Newly explored natural fibers with having adequate properties that can fulfill the present demand in some extend.

Therefore, we are actively engaged in exploring new sources of plant-based natural fiber and dyes from such parts of a plant which are reproducible e.g. leaves, bark of branches etc. [7, 8]. As a part of our on-going research we report here a new high-quality cellulose rich natural fiber that extracted from the bark of branches of jack tree using a cheap and easy extraction technique. Jack tree (*Artocarpus heterophyllus* Lam) which is also known as Jackfruit belongs to the Moraceae family. The jack tree is widely cultivated in tropical regions specially in South and Southeast Asian countries, in the Caribbean, Latin America and some parts of Africa. Jack tree is an evergreen tree that has a relatively short trunk with a dense treetop and can live about hundred years. Though trees generally take a long time to mature, jack trees are comparatively fast growers. In about three or four years after planting, one can expect the jackfruit tree to produce harvestable fruits. Matured jack trees can reach the heights of 10–20 m with trunk diameters of 30–80 cm. Its bark is reddish-brown and smooth. A milky juice is released in the event of injury to the bark. It produces the largest edible fruit of all trees and a good source of vitamin and minerals. A full-grown tree has many branches and newer branches sprout every year. It is reported that different parts of the jackfruit tree have various medicinal properties [9, 10, 11, 12]. It is also reported that Jackfruit peel is used as a source of cellulose nanoparticles and activated carbon synthesis [13, 14].

Since, a jack tree is long-lived and considerably huge with too many reproducible branches so, it is possible to collect a good amount of cellulosic fiber from the bark of branches repeatedly for many years without damaging the tree. A reproducible source containing rich cellulosic fiber is very significant and sustainable. So, the new fiber from the bark of jack tree branches would be a worthwhile addition to the pool of natural fibers.

## 2 Experimental

### 2.1 Extraction of fiber

**Figure 1:**
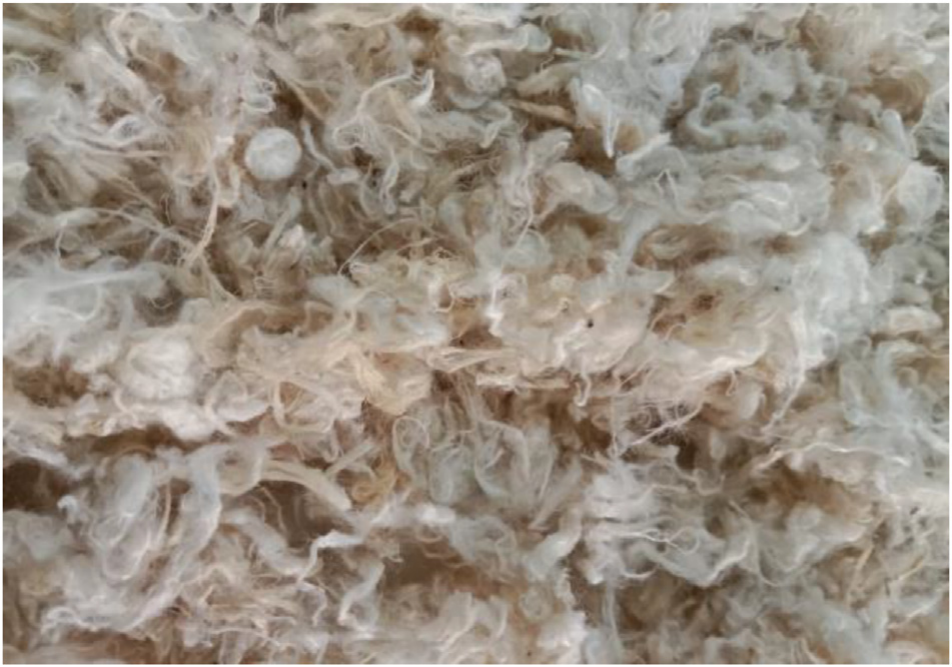
Photograph of fibers extracted from the bark of jack tree branches.

The fresh and young branches of jack tree were collected from the northern part of Bangladesh. The bark of these branches was separated manually and immersed in fresh water to allow microbial degradation for a period of 10–12 days. After that, the fiber was separated by hand and washed several times with clean water to remove unwanted ingredients. Finally, the fibers were dried at room temperature for a week and used it directly for analysis without performing any chemical treatment. The photograph of extracted fibers is shown in Figure 1.

### 2.2 Chemical composition

The chemical composition of the fiber collected from the bark of jack tree branches i.e. its cellulose, hemicellulose, lignin, extractive and ash contents were measured according to TAPPI standard test procedures. The extractive percentage was estimated by Soxhlet extraction technique using ethanol-toluene mixture as a solvent (TAPPI T204 om-88). The extractive free fibers were used for the preparation of hollo-cellulose using NaClO_2_ solution and then α-cellulose content was measured by treating the obtained hollo-cellulose with NaOH solution (TAPPI T203 om-93). For measuring the lignin percentage, Klason technique was applied (TAPPI T211 om-83). The ash content was measured in accordance with TAPPI (T211 os-76) test method. Two replications were done for each compositional analysis and the average is counted [15, 16].

### 2.3 XRD analysis

The crystalline property of the fiber was measured using X-ray diffractometer (Smart Lab SE, Regaku, Japan) with X-ray generator settings of 50 mA current and 40 kV voltages. The fiber pellet sample was used for X-ray diffraction analysis. The diffraction intensity of CuK_α_ (1.54 Å) radiation was recorded between 10° and 40° (2*θ* angle range) with a scanning speed 15°/min in step width of 0.01°. The crystallinity index (*CI*) in the cellulosic materials was determined according to the peak height technique established by the subsequent Segal empirical equation [17].

(1) $$CI\%=\frac{I_{200}-I_{am}}{I_{200}}x100$$

Where, I_200_ is the maximum intensity of crystalline phase at 2*θ* = 22.729° and I_am_ is the minimum intensity calculated by height of the valley of the minimum between the peaks at around 2*θ* = 18.9° [18, 19].

The crystalline size was calculated using Scherrer's formula [20].

(2) $$CS=\frac{K\lambda }{\beta \;\cos\;\theta }$$

Where, K = 0.89 is the Scherrer's constant, *β* is the peak's full width at half maximum in radians, *λ* is the wave length of radiation and *θ* is the Bragg angle corresponding to half of the 2*θ* angle (see Figure 1).

**Figure 2:**
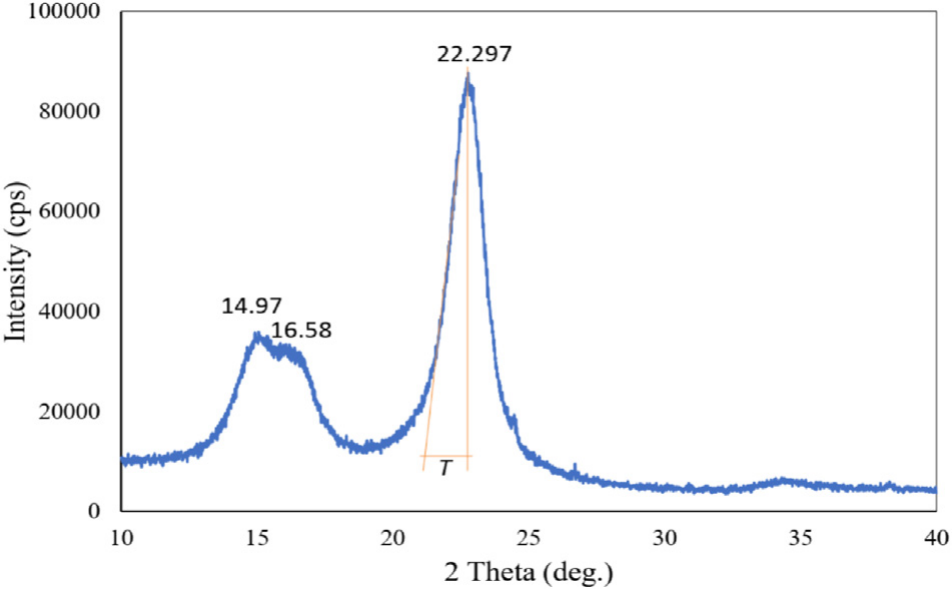
X-ray diffraction pattern of the fiber.

The MFA (microfibrillar angle) of the fibers was calculated from XRD data. The angular separation ‘*T*’ as shown in Figure 2 which is the distance between the equator and the point where the tangent at the point of inflexion of the intensity curve cuts the zero intensity axes. The mean MFA of the fibers was calculated from this value of ‘*T*’ using Yamamoto relationship [21].

(3) Mean MFA = 1.575 × 10^−3^*T*^3^ – 1.431 × 10^−1^*T*^2^ + 4.693*T*–36.19

### 2.4 FTIR analysis

Fourier transform infrared (FTIR) spectra of the fiber were measured using an FTIR spectrophotometer (PerkinElmer Spectrum Two, Beaconsfield, UK). For this measurement, the sample was ground into powder form and pressed with KBr into a pellet. The spectra were recorded in the region 400–3900 cm^−1^ at a resolution of 2 cm^−1^ in the transmittance mode.

### 2.5 TGA and SEM analysis

The thermal stability of the fiber was performed with a thermogravimetric analyzer (Hitachi TG-DTA 7200, Japan) using an aluminum pan at a heating rate of 10 °*C min*^−1^ under N_2_ gas flow of 100 mL min^−1^. For this experiment, 12.674 mg of dried sample was used. Temperature inside the test chamber of the thermal analyzer was increased gradually at 10 °C per minute rate from 32.6 °C to 565.44 °C.

The SEM analysis was carried out using Carl ZEISS EVO 15 scanning electron microscope. The specimens were coated with a 10 nm of gold layer using a 15-kV beam sputter coater to avoid the accumulation of electric charges during analysis. Energy dispersive spectrometer (EDS) is used for the elemental analysis which is attached on the Scanning Electron Microscope.

### 2.6 Tensile strength analysis

Tensile properties of the fiber, in terms of strength and elongation were measured using USTER HVI 1000 instrument (HVI SW Version 3.4.1.71). The instrument offers very precise and reliable data through its computer-controlled calibration and diagnostics facilities. The fiber sample was run three times in HVI machine and the average was taken. The standard deviation was found ±0.1 and ±0.44 for the strength and elongation respectively. To calculate the fiber length, 20 fibers were taken from extracted fibers. The length was measured by standard ruler and the average was counted.

### 2.7 Density measurement

The density of the fiber was determined by pycnometer where water immersion technique was used to find the density of the fiber. Then, the density was calculated according to the following equation [22].

(4) $$\rho_{f}=\frac{\left(m_{2}-m_{1}\right)}{\left(m_{3}-m_{1}\right)-\left(m_{4}-m_{2}\right)}\;\rho_{w}$$

Where *ρ*_*f*_ is the density of fiber in g/cc, *ρ*_*w*_ is the density of water (0.997 g/cc at 25 °C), m_1_ is the mass of empty pycnometer, m_2_ is the mass of pycnometer with fiber, m_3_ is the mass of water filled pycnometer and m_4_ is the mass of pycnometer filled with fiber and water.

## 3 Results and discussion

### 3.1 Extraction process

Simple water retting process is good enough for the extraction of fibers from the bark of jack tree branches. The water retting process was carried out by immersing the bark in fresh water for 10–12 days. There are several methods of extraction which are employed to collect the fibers from different parts of the plant such as water retting, mechanical, alkali treatment etc. In water retting process, the action of microorganism rot away non-cellulosic part from the bark and makes it easy to separate the fibers. It is the cheapest process and environmentally friendly. Unfortunately, this natural process is not suitable for all the extraction processes. Therefore, alkali like NaOH in different concentration at the temperature of 95–100 °C is used in many cases for the extraction purpose (Table 1) which is hazardous, costly and corrosive to the used equipment due to the strong alkaline nature of NaOH. We are fortunate that water retting is good enough for collecting the high-quality cellulosic fibers from the jack tree branches, which definitely add some extra values to its production process. Some non-conventional methods are also being used for extracting the natural cellulosic fibers from various sources [23, 24, 25, 26].

**Table 1:** Comparison of chemical constituents of jack tree fiber with some common natural fibers.

Fiber type	Cellulose (%)	Hemi- Cellulose (%)	Lignin (%)	Extraction process	Density (g/cc)	Crystallinity Index (%)	References
**Jack tree fiber**	**79.32**	**8.01**	**6.77**	**Water retting**	**1.05**	**86**	**Present work**
Flax	62–71	16–18	3–4.5	--	1.5	80	[22]
Hemp	67–75	16–18	3–5	--	1.47	88	[22]
Star jasmine	62.7	14.5	7.6	Water retting and then Enzyme retting	1.398	87.68	[22]
*Cissus quadrangularis* root	77.17	11.02	10.45	Water retting	1.51	56.6	[27]
*Cissus quadrangularis* stem	82.73	7.96	11.27	Water retting	1.22	47.15	[27]
Jute	64.4	12	11.8	Water retting	1.3	71	[27]
Sorghum stems	65	--	6.5	2% NaOH, 95 °C	--	--	[28]
*Prosopis juliflora* bark	61.65	16.14	17.11	Water retting	0.581	46	[29]
*Furcraea foetida*	68.35	11.46	12.32	Water retting	0.778	52.56	[30]
*Acacia Planifrons*	73.1	9.41	12.04	Water retting	0.660	65.38	[31]
*Artisdita hystrix*	59.54	11.35	8.42	--	0.540	44.85	[31]
*Albizia amara*	64.54	14.32	15.61	Water retting	1.043	63.78	[31]
Saharan aloe vera cactus	60.2	14.2	13.7	Water retting	1.325	52.6	[32]
	67.4	8.2	13.7	5% NaOH	1.623	56.5	
Cornhusks	80–87	--	--	0.5N NaOH, 95 °C	--	48–50	[33]
Cotton stalk	79	--	13.7	2N NaOH, boiling temperature	--	47	[34]

### 3.2 Chemical analysis

The chemical composition of the fiber was determined by TAPPI standard test procedures and the test values of the common constituents are: α-cellulose 79.32%, hemicellulose 8.01%, lignin 6.77%, ash 3.58%, extractives 2.32% and moisture content 7.4%. Jack tree fiber showed higher cellulose content associated with lower fractions of lignin and hemicellulose. Comparisons of these values with the respective values of some other common natural fibers are presented in Table 1. The cellulose content of jack tree fiber is higher compared to many other known natural fibers. It is very much encouraging that such high cellulose content fiber obtained without doing any chemical treatment.

The composition of the natural fibers has great impact on their properties. High cellulose content encourages high value applications and relatively easier to process them for various applications. It can enhance the quality of the materials such as mechanical properties, biodegradability, thermal stability, crystalline properties, resistance to hydrolysis etc. Moderate amount of hemicellulose and lignin content can contribute to control the fiber bundle and the rigidity that make them suitable for high value composite application.

### 3.3 XRD analysis

The X-ray diffraction pattern of the fiber is shown in Figure 2. As seen in the Figure, there are three main diffraction peaks at 2θ = 14.97°, 16.58° and 22.729° corresponding to the 1–10, 110 and 200 planes respectively. These three characteristic peaks are usually observed for cellulose-I_*β*_ found in natural fibers where cellulose exists in parallel strands with inter-sheet hydrogen bonding.

I_*α*_ and I_*β*_ - these two types cellulose are found in nature. Only the I_*β*_ cellulose is available in the higher plants therefore, it has been taken into consideration in this analysis. On the other hand, I_*α*_ is found in bacterial and alga celluloses [35]. Cellulose I_*β*_ is made of paralleled hydrogen-bonded sheets that stack with an alternating shear stabilized by van der Waals interactions. The I_*α*_ cellulose exists as triclinic lattice with one chain per unit cell whereas, the I_*β*_ cellulose exists as monoclinic lattice with two chains per unit cell [36, 37]. The measured crystallinity index of jack tree fiber is found 86% according to the Segal formula which is comparable to the fibers extracted from hemp (88%), flax (80%) and star jasmine (87.68%) but higher than other natural cellulosic fiber [22, 38]. High crystallinity means good strength of the fiber and they can provide better reinforcement for composite materials. The crystallinity has also an effect on moisture absorption of the fibers, since amorphous regions can absorb more water. The thermal decomposition of natural fibers is shifted to higher temperatures with an increase in the cellulose crystallinity and crystallite size (CS) [39]. Smaller crystal size means more surface area and more accessible to water and chemicals. The CS of the jack tree fiber was found 5.19 nm that almost similar to cotton. The crystalline sizes of some natural fibers are: cotton 5.5 nm, corn stalk fibers 3.8 nm, flax/linen/jute fibers 2.8 nm, ramie fibers 16 nm, Raffia textiles 32 nm [33, 40, 41].

### 3.4 FTIR analysis

**Figure 3:**
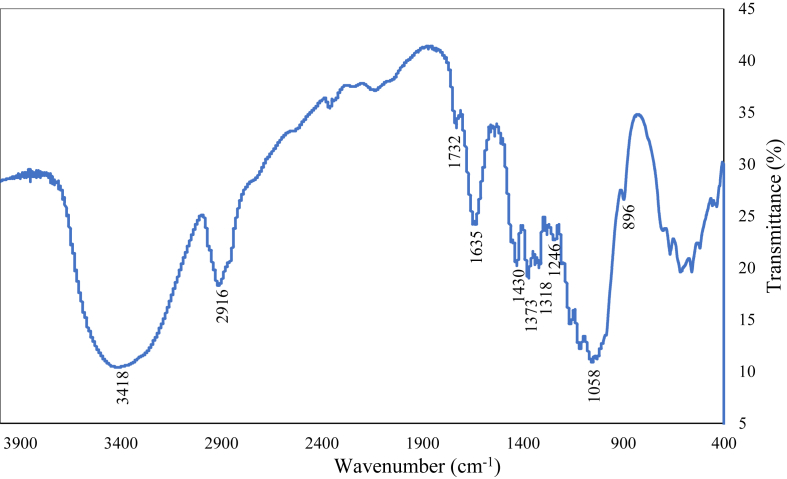
FTIR spectrum of the fiber.

The FTIR spectrum of the fiber is shown in Figure 3. The spectrum showed well-defined peaks at 3418, 2916, 1732, 1635, 1535, 1430, 1373, 1318, 1280, 1246, 1163, 1114, 1058, 896 cm^−1^. A broad peak at 3418 cm^−1^ is corresponding to the stretching vibration of hydrogen bonded hydroxyl group. The peak at 2916 cm^−1^ is for C–H stretching vibration of cellulose and hemicellulose [2, 42]. The peaks at 1635 and 1732 cm^−1^ are corresponding to the carbonyl groups (C=O) of lignin and hemicellulose [29, 43, 44]. The strong peak at around 1635 cm^−1^ is observed for water absorption for natural cellulose fiber [32, 45, 46]. A very low intensity peak at 1535 cm^−1^ is corresponding to the C=C groups of lignin. The peak at 1430 cm^−1^ may be due to the bending vibration of CH_2_ groups of cellulose. The presence of peak at 1373 cm^−1^ is associated to the C–H bending vibration. The peak at 1318 cm^−1^ is due to the O–H bending [47].

The C–O stretching vibration of acetyl group in lignin is at 1246 cm^−1^. Peaks at around 1100-1200 cm^−1^ are attributed to C–O–C vibration for cellulose and hemicellulose content. The peak at 1058 cm^−1^ is suggested for C–OH vibration and 896 cm^−1^ is corresponded to *β*-glycoside linkage [22, 48]. The peak patterns of FTIR support the presence of high cellulose content in this fiber [46].

### 3.5 Thermogravimetric analysis

**Figure 4:**
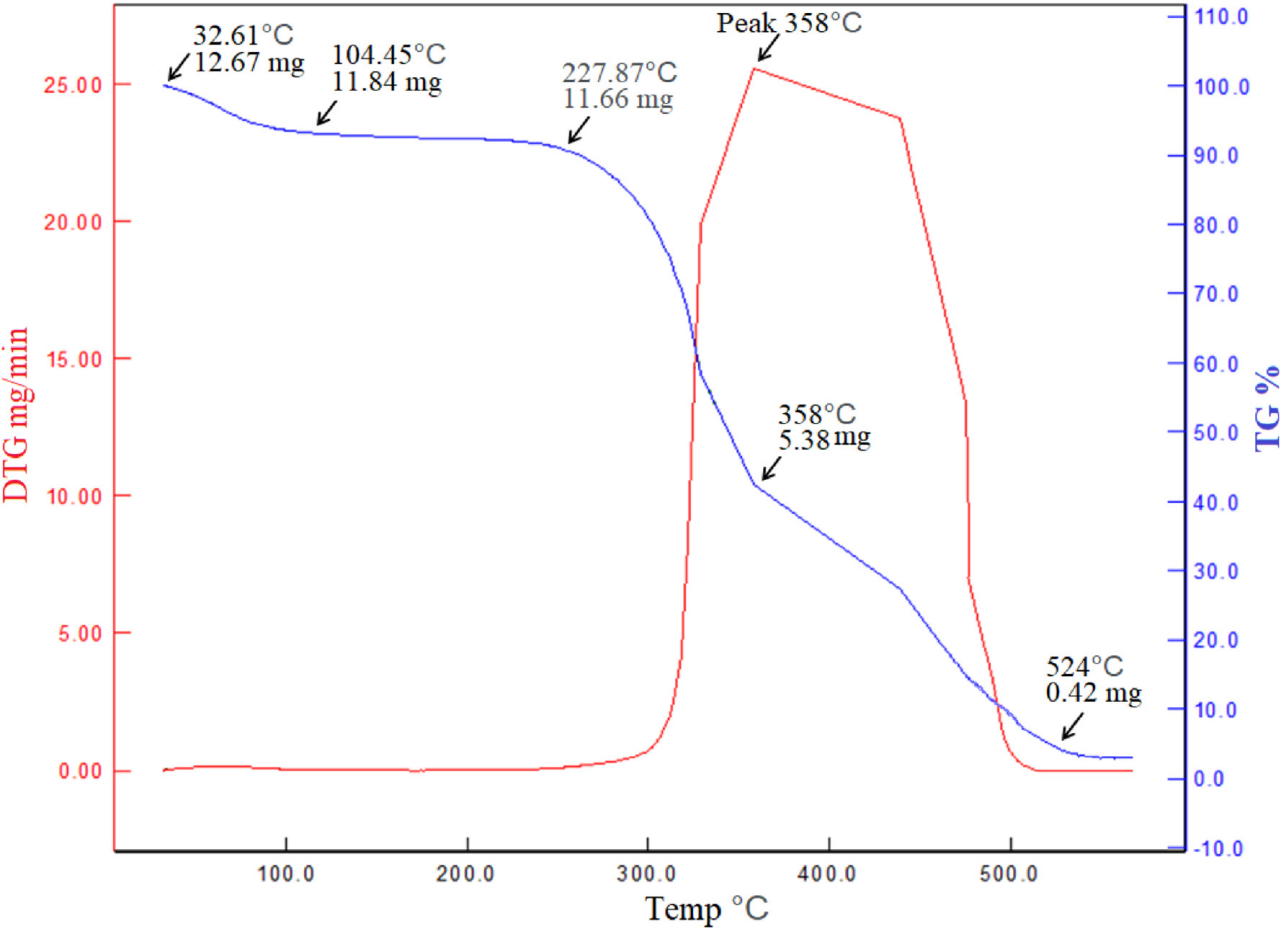
Thermo gravimetric analysis curve of the fiber.

The thermogravimetric analysis and derivative thermogravimetric (TGA and DTG) of jack tree fiber is shown in Figure 4. The initial weight loss happened due to the evaporation of moisture on the surface of fiber at around 100 °C where the fiber suffered 6% mass change. Then the fiber was thermally stable up to 228 °C (mass change 1.5% only, 100–228 °C).

Main three components cellulose, hemicellulose and lignin control the thermal stability and their stability order is hemicellulose < cellulose < lignin. A major decomposition was occurred after 228 °C. This degradation stage was involved for hemicellulose components. The maximum degradation peak is at 358 °C which was mainly due to the degradation nature of α-cellulose (50 % mass change, 228–358 °C). Cellulose rich and high crystalline structures increase the degradation temperatures [49, 50]. The next stage (after 358 °C) is mainly involved for cellulose and lignin decomposition. Similar degradation behaviors were observed in various reported cellulose based natural fibers [40, 44, 47]. The measurement was done up to 565 °C where 97% decomposition of the fiber was occurred. This result is consistent with the composition of the fiber.

Cellulose, hemicellulose and lignin contents of natural fibers have significant influence on their mechanical and physical properties [51, 52]. The presence of more crystalline cellulose in a fiber means it possesses the higher tensile strength and higher thermal stability [53]. The initial thermal degradation behavior and the moisture content of a fiber are highly influenced by hemicellulose [51]. The natural fibers which contain a high amount of hemicellulose should absorb more moisture and degrade at a lower temperature. Similarly, high quantities of extractives can facilitate the fiber degradation at a lower temperature [52]. Thus, it may be possible to estimate the degradation pattern of natural fibers based on their chemical composition. The hemicellulose and lignin contents of jack tree fiber are comparatively low than many common natural fibers (Table 1). This is the reason why the jack tree fiber shows high thermal stability.

### 3.6 SEM and EDS analysis

**Figure 5:**
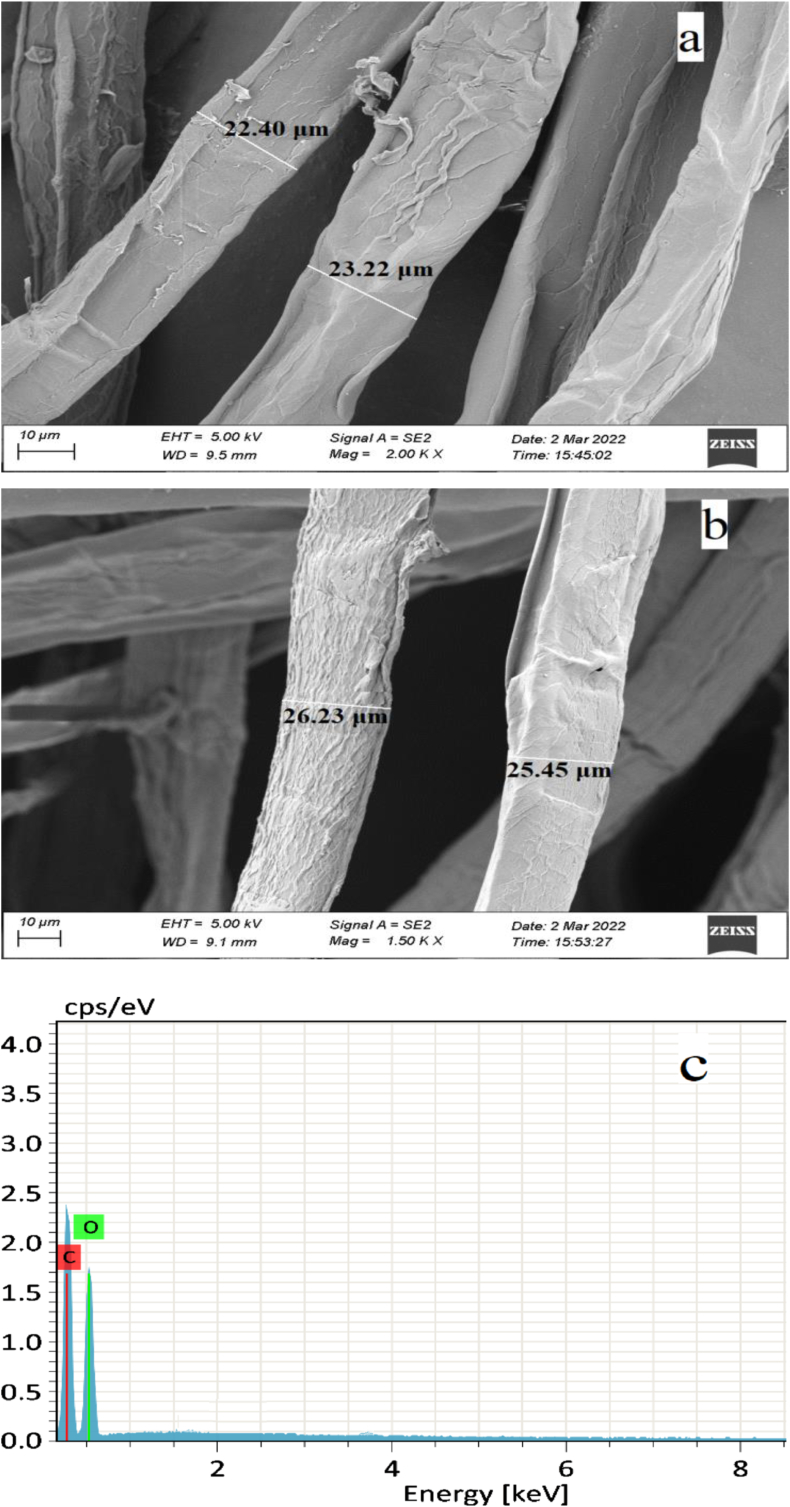
(a) & (b) SEM images of the fiber surface; (c) EDS spectrum.

The morphological features of jack tree fiber are shown in Figure 5.

Without any chemical treatment, the outer layer of the fiber has a slight smoothness [34, 48]. However, mostly it has rough and non-uniform outer surface. The outer layer of the fiber is composed of wax, lignin and hemicellulose that are recognized as binding materials on the surface of the fiber. The surface roughness contributes for increased contact surface area to ensure better adherence of the fiber with the matrix in composite application. The average length and diameter were found 18.5 mm and 23.2 μm respectively. The diameter of the fiber is very similar with cotton, linen and jute fiber [33]. The elemental analysis was also performed by EDS spectra that showed two main peaks for carbon and oxygen. It presented the amount of element distributed on the surface of the fiber in terms of weight (50.42% C and 49.58% O) and atomic percent (57.5% C and 42.5% O) and the results are very much consistent with the results of natural cellulosic fibers [54, 55, 56].

### 3.7 Tensile strength analysis

**Figure 6:**
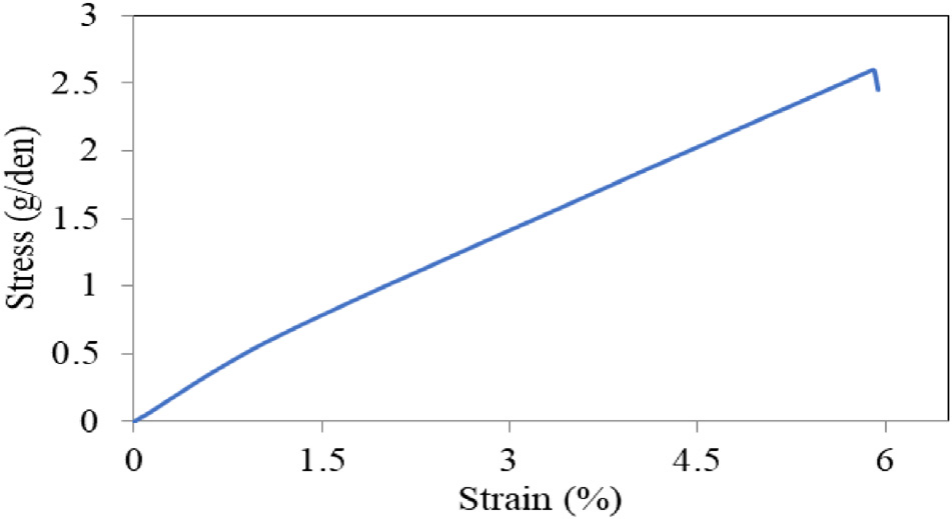
Tensile stress-strain graph of the fiber.

The stress- strain curve of the fibers extracted from the bark of jack tree branches is given in Figure 6. Almost a linear region and sudden drop in stress value are observed from this curve. The sudden failure is mainly due to the amorphous constituents present in the fiber which cause the rapture. The fibers possess moderate tensile strength and good elongation properties and are capable of withstanding pulling forces. The mean strength and elongation of the fiber were found 2.6 g/denier and 5.9% respectively. The breaking tenacity is near to that of cotton, sorghum, corn husk, flax, jute (Table 2). However, breaking elongation is near to cotton but higher than many other natural fibers. The value of ‘*T*’ and MFA are 1.6° and –29° respectively. The negative symbol in the MFA value exhibit that the cellulose microfibrils are oriented on the backside cell wall of the plant fibers. The MFA of the fibers are very similar to the mean MFA of *Cocos nucifera* fibers [57]. The tensile strength also depends on microfibrillar angle. Larger microfibrillar angles can cause lower strength, more ductility and more strain rate [43]. However, these parameters may vary on source, age of the plant, fiber extraction process, plantation environment, test conditions etc.

**Table 2:** Comparison of mechanical properties.

Fiber type	Tenacity (g/denier)	Elongation (%)	References
**Jack tree fiber**	**2.6**	**5.9**	**This work**
Cotton	2.7–3.5	6–9	[34]
Cotton stalk fibers	2.9	3	[34]
Cornhusks	2.7	15.3	[28]
Corn stems	2.2	2.2	[28]
Flax	2.6–2.7	1.2–1.6	[44]
Sorghum Leaf and Stem Fibers	2.3–2.4	2.6	[28]
Jute	2.8	1.8	[44]

### 3.8 Density analysis

The density of the fiber is also a very important factor for composite application. A comparison with other fibers is shown in Table 1. The determined density of jack tree fiber is 1.05 g/cc, which is much lower than that of many reported fibers such as cotton, sisal, ramie, flax, jute etc. [22, 58]. The low-density fibers are very suitable for reinforcement in composite especially for light weight application.

## 4 Conclusion

A new natural cellulosic fiber was extracted from bark of jack tree branches by using simple water retting process and characterized by various standard techniques and analyses. The chemical composition of the fiber shows that it could be an excellent source of cellulose rich natural fiber (79.32%), which is comparable to cotton and superior than other reported natural fibers. XRD and FTIR results reveal that the fiber has similarities with cotton. Good morphology was observed by SEM analysis. The density of the fiber is relatively low compared to other natural fibers indicating its suitability for light weight application. Thermogravimetric analysis and derivative thermogravimetric (DTG) indicate that the fiber is thermally stable up to 228 °C and the major degradation is occurred at the range 228–358 °C. This degradation pattern indicates that the fiber is rich in cellulose content and high crystalline structures. Moderate strength and good elongation properties are observed in this fiber. The important properties of the new fiber indicate its suitability in some special applications such as reinforcement in composite, cellulose nano-materials, activated or conductive carbon, biomaterials etc. Further research is in progress to investigate the suitability of this fiber as reinforcement materials for bio-composite.

## Declarations

### Author contribution statement

Shahin Hossain: Conceived and design the experiments; Performed the experiments; Analyzed and interpreted the data; Wrote the paper.

M. Abdul Jalil: Analyzed and interpreted the data.

Tarikul Islam, Md Mostafizur Rahman: Analyzed and interpreted the data; Contributed reagents, materials, analysis tools or data.

### Funding statement

This research did not receive any specific grant from funding agencies in the public, commercial, or not-for-profit sectors.

### Data availability statement

Data included in article/supplementary material/referenced in article.

### Declaration of interests statement

The authors declare no conflict of interest.

### Additional information

No additional information is available for this paper.
